# Apoptotic activity is increased in parallel with the metaplasia–dysplasia–carcinoma sequence of the bronchial epithelium

**DOI:** 10.1038/sj.bjc.6690159

**Published:** 1999-02

**Authors:** U Törmänen, K Nuorva, Y Soini, P Pääkkö

**Affiliations:** Department of Pathology, University of Oulu, Oulu, Finland; Department of Pathology, Central Finland Health Care District, Jyväskylä, Finland; Oulu University Hospital, Oulu, Finland

**Keywords:** apoptosis, cell proliferation, p53, bcl-2-family, bronchial dysplasia

## Abstract

A high level of apoptotic activity and an independence of apoptosis from the expression of p53 and bcl-2 have been observed in non-small-cell lung carcinoma. We examined 44 samples of normal, metaplastic and premalignant (i.e. mild, moderate and severe dysplasias and carcinoma in situ) bronchial epithelia to evaluate whether differences in the apoptotic activity could already be seen in the stages preceding squamous cell carcinoma of the lung (SQCLC). Apoptotic cells and bodies were visualized by 3′ end labelling. The expression of *p53* and members of the *bcl-2* gene family, such as *bcl-2*, *bax* and *mcl-1*, were determined immunohistochemically with specific antibodies. The relative number of apoptotic cells and bodies [apoptotic index (AI%)] was already increased threefold as the normal bronchial epithelium changed to squamous metaplasia, and the AIs of the dysplastic lesions were about four times higher than those of the normal epithelium. Apoptosis was significantly associated with cell proliferation, as determined by proliferating cell nuclear antigen (PCNA) immunohistochemistry. However, the extent of apoptosis did not correlate with the expression of p53, bcl-2, bax and mcl-1. We conclude that, in the metaplasia–dysplasia–carcinoma sequence in the lung, the elevation of the AI% is an early event associated with cell proliferation activity, but is independent of the expression of p53, bcl-2, mcl-1 and bax. © 1999 Cancer Research Campaign


*British Journal of Cancer* (1999) **79**(5/6), 996–1002
© 1999 Cancer Research Campaign
Article no. bjoc.1998.0159

### Apoptotic activity is increased in parallel with the metaplasia–dysplasia–carcinoma sequence of the bronchial epithelium

**U Törmänen[1], K Nuorva[2], Y Soini[1,3] and P Pääkkö[1,3]**

[1]Department of Pathology, University of Oulu, Oulu, Finland; [2]Department of Pathology, Central Finland Health Care District, Jyväskylä, Finland; [3]Oulu University Hospital, Oulu, Finland

**Summary** A high level of apoptotic activity and an independence of apoptosis from the expression of p53 and bcl-2 have been observed in non-small-cell lung carcinoma. We examined 44 samples of normal, metaplastic and premalignant (i.e. mild, moderate and severe dysplasias and carcinoma in situ) bronchial epithelia to evaluate whether differences in the apoptotic activity could already be seen in the stages preceding squamous cell carcinoma of the lung (SQCLC). Apoptotic cells and bodies were visualized by 3′ end labelling. The expression of *p53* and members of the *bcl-2* gene family, such as *bcl-2*, *bax* and *mcl-1*, were determined immunohistochemically with specific antibodies. The relative number of apoptotic cells and bodies [apoptotic index (AI%)] was already increased threefold as the normal bronchial epithelium changed to squamous metaplasia, and the AIs of the dysplastic lesions were about four times higher than those of the normal epithelium. Apoptosis was significantly associated with cell proliferation, as determined by proliferating cell nuclear antigen (PCNA) immunohistochemistry. However, the extent of apoptosis did not correlate with the expression of p53, bcl-2, bax and mcl-1. We conclude that, in the metaplasia–dysplasia–carcinoma sequence in the lung, the elevation of the AI% is an early event associated with cell proliferation activity, but is independent of the expression of p53, bcl-2, mcl-1 and bax.

**Keywords:** apoptosis; cell proliferation; p53; bcl-2-family; bronchial dysplasia

Squamous cell carcinoma of the lung (SQCLC) is generally associated with smoking and is preceded by clinically and histologically definable preinvasive lesions of the bronchial epithelium (Auerbach et al, 1957, 1962, 1979). Squamous cell metaplasia, developing from basal cell hyperplasia, is the earliest recognizable morphological, but not yet neoplastic, change. Mild, moderate and severe dysplasias and squamous cell carcinoma in situ are already premalignant, but still non-invasive lesions (World Health Organization, 1981). The malignant phenotype of invasive carcinoma is believed to develop as a result of aberrations in the expression and function of oncogenes, proto-oncogenes and tumour-suppressor genes (e.g. *bcl-2* and *p53*), leading to a growth advantage of the neoplastic cells. *p53* and the genes of the *bcl-2* family, such as *bcl-2*, *bax* and *mcl-1*, are regulatory genes of apoptosis, an event in which single cells die following a highly defined, predetermined programme. During this event, several morphological changes lead to the formation of membrane-bound apoptotic bodies, which are then phagocytosed by neighbouring cells (Kerr et al, 1972).

Mutations in the *p53* tumour-suppressor gene are found in 37% of all human malignancies and in 60% of lung carcinomas (Greenblatt et al, 1994). Aberrant expression of p53 has also been detected in preinvasive lesions of the bronchial epithelium (Sozzi et al, 1992; Sundaresan et al, 1992; Vähäkangas et al, 1992; Bennett et al, 1993; Nuorva et al, 1993; Hirano et al, 1994; Walker et al, 1994), in which it is associated with the severity of dysplasia (Sundaresan et al, 1992; Bennett et al, 1993; Nuorva et al, 1993; Hirano et al, 1994; Walker et al, 1994).

The effects of the ever-expanding *bcl-2* gene family on apoptosis are under intensive research. The first member of this group was the *bcl-2* proto-oncogene, which was first identified in follicular lymphomas (Tsujimoto et al, 1984; Bakhshi et al, 1985) and was later found to be an inhibitor of apoptosis induced by a wide variety of stimuli (Reed, 1994). In normal skin, bcl-2 is expressed in the basal epithelial cells (Hockenbery et al, 1991; Lu et al, 1993). In bronchial dysplasias, bcl-2 expression can be detected throughout the epithelial layer, and the irregularity of the staining pattern increases concurrently with the degree of dysplasia (Walker et al, 1995). *bax*, a member of the *bcl-2* gene family, produces a protein with approximately 21% amino acid sequence homology with *bcl-2* (Oltvai et al, 1993). When overexpressed, bax forms homodimers and a cell death-promoting signal is accelerated. In contrast, when bcl-2 is in excess, it heterodimerizes with bax and cell death is repressed (Oltvai et al, 1993). Thus, it has been suggested that the ratio of bcl-2 to bax determines the susceptibility of a cell to apoptosis (Oltvai et al, 1993). Mcl-1, which displays functional similarity to bcl-2, is capable of binding to bax and suppressing bax-induced cytotoxicity (Bodrug et al, 1995).

Recently, it was shown that members of the *bcl-2* family regulate apoptosis through their ability to alter the mitochondrial membrane potential and to form ion channels (Kroemer, 1997;

*Received 2 May 1998*
*Revised 27 May 1998*
*Accepted 3 June 1998*

*Correspondence to:* P Pääkkö, University of Oulu, Department of Pathology, Kajaanintie 52 D, FIN-90220 Oulu, Finland

Minn et al, 1997; Yang et al, 1997). This leads to the liberation of caspase-activating substances, such as cytochrome c (CK) and apoptosis-inducing factor (AIF), from the mitochondria into the cytosol causing apoptosis of the cell. The proapoptotic bax has been shown to induce the liberation of such caspase-activating substances, whereas the antiapoptotic bcl-2 and bcl-$x_L$ inhibit it (Kroemer, 1997; Minn et al, 1997; Yang et al, 1997).

A high level of apoptotic activity in tumours is generally considered to signify slower tumour growth and a better prognosis. In our previous work, however, enhanced apoptosis in non-small-cell lung carcinoma (NSCLC) was associated with shortened survival (Törmänen et al, 1995). In addition, the relative number of apoptotic cells and bodies was independent of the expression of p53 and bcl-2 (Törmänen et al, 1995). In human colorectal tubular adenomas, apoptotic cells are seen more frequently in high- than in low-grade dysplasias (Arai and Kino, 1995). In the light of these observations, it was of interest to examine whether there is a trend towards increased apoptosis in the premalignant lesions preceding SQCLC and whether aberrations in the expression of apoptosis-regulating genes, such as *p53*, *bcl-2*, *bax* and *mcl-1*, can already be seen at this stage. We analysed the extent of apoptosis in 44 bronchial tissue samples representing normal bronchial epithelium, squamous metaplasia, bronchial epithelial dysplasia and carcinoma in situ.

### MATERIALS AND METHODS

### Case selection and classification of lesions

Forty-four formalin-fixed samples containing premalignant, non-invasive, bronchial lesions were collected from the archives of the Departments of Pathology, Oulu University Hospital, Finland, and Central Finland Health Care District. All specimens were histopathologically re-evaluated according to the World Health Organization Histological Typing of Lung Tumours (1981), and representative tissue samples were chosen for further studies. The bronchial epithelium was normal in 14 cases, squamous metaplasia was found in eight cases, mild epithelial dysplasia in three, moderate dysplasia in eight and severe dysplasia in six cases. The lesions in five samples were graded as squamous cell carcinoma in situ.

### 3′ End labelling of apoptotic DNA fragments

Apoptotic cells and bodies in tissue sections were detected using an ApopTag in situ Apoptosis Detection Kit (Oncor, Gaithersburg, MD, USA). The instructions laid out by the manufacturer were followed with a few modifications, as described previously (Törmänen et al, 1995). A cell was defined as apoptotic if the whole nuclear area was positively labelled, whereas apoptotic bodies were identified as small, positively labelled, globular fragments. All positively labelled cells and bodies fulfilled the morphological criteria for apoptosis as described by Kerr et al (1972), i.e. condensation of the nucleus, cell shrinkage, cytoplasmic budding to form membrane-bound fragments, and detachment from surrounding cells.

The number of apoptotic cells and bodies in the epithelial tissue was counted in ten high-power fields (HPFs; objective ×40, field diameter 400 μm) when possible. In some specimens, the amount of epithelium was not sufficient; in these cases, a minimum of four HPFs were studied. Apoptotic bodies likely to be originating from the same apoptotic cell were recorded as one apoptotic body. The quantity of apoptotic cells and bodies, or apoptotic index (AI%), is expressed as a percentage of the whole epithelial cell population.

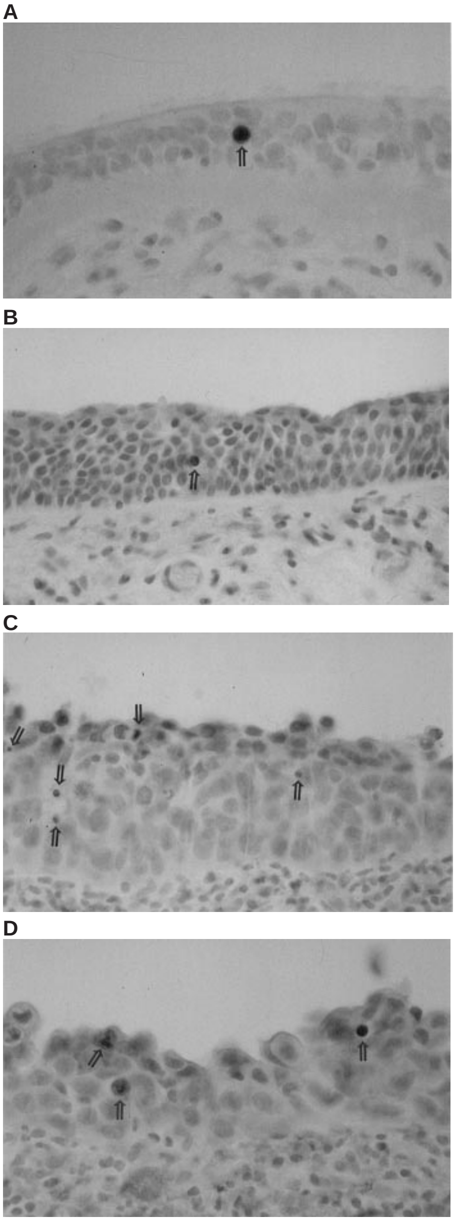

**Figure 1** The extent of apoptosis in normal bronchial epithelium and in the metaplasia–dysplasia–carcinoma sequence of the bronchus. 3′ End labelling of apoptotic DNA. (**A**) One apoptotic cell (arrow) in normal bronchial epithelium. (**B**) One apoptotic cell (arrow) in metaplastic epithelium. (**C**) Four apoptotic bodies (arrows) in a moderately dysplastic area of the epithelium. (**D**)Three apoptotic cells (arrows) in a squamous cell carcinoma in situ

### Immunohistochemistry for p53

The immunohistochemistry for p53 was performed as described previously by Törmänen et al (1995), using a polyclonal antibody CM-1 (Novocastra Laboratories, Newcastle upon Tyne, UK) at a dilution of 1:1000.

© Cancer Research Campaign 1999

**Table 1** The extent of apoptosis in normal and metaplastic bronchial epithelia, bronchial dysplasias and squamous cell carcinoma in situ of the lung

| | Apoptotic cells (%) mean (range) | Apoptotic bodies (%) mean (range) | Apoptotic index (%) mean (range) | Number of cases studied |
|---|---|---|---|---|
| Normal epithelium | 0.18 (0.00–0.39) | 0.16 (0.00–0.45) | 0.34 (0.00–0.69) | 14 |
| Squamous metaplasia | 0.34 (0.00–0.88) | 0.59 (0.00–1.00) | 0.92 (0.40–1.57) | 8 |
| Mild dysplasia | 0.66 (0.27–1.11) | 0.79 (0.41–1.13) | 1.45 (0.68–1.94) | 3 |
| Moderate dysplasia | 0.47 (0.00–1.11) | 1.19 (0.09–3.60) | 1.66 (0.18–4.40) | 8 |
| Severe dysplasia | 0.64 (0.32–1.29) | 0.73 (0.20–1.34) | 1.37 (0.52–2.63) | 6 |
| Carcinoma in situ | 0.51 (0.21–0.83) | 0.98 (0.21–2.48) | 1.49 (0.63–3.26) | 5 |

Apoptotic indices were significantly higher in preinvasive bronchial lesions than in normal and metaplastic epithelia ($P < 0.001$).

**Table 2** Distribution of p53 positivity in normal, metaplastic and dysplastic bronchial epithelia

| | Immunoreactivity for p53 | | | | | | | |
|---|---|---|---|---|---|---|---|---|
| Type of epithelium | Negative | <1% | 1–5% | 6–10% | 11–40% | >40% | NA | Total |
| Normal | 14 | | | | | | | 14 |
| Squamous cell metaplasia | 7 | 1 | | | | | | 8 |
| Mild dysplasia | 3 | | | | | | | 3 |
| Moderate dysplasia | 6 | | | 1 | 1 | | | 8 |
| Severe dysplasia | 4 | | 2 | | | | | 6 |
| Carcinoma in situ | | | | 2 | 1 | 1 | 1 | 5 |
| Total number of sections | 34 | 1 | 2 | 3 | 2 | 1 | 1 | 44 |

Abnormal accumulation of p53 protein (>1% of p53-positive nuclei) was more often observed in mild, moderate and severe dysplasias and carcinoma in situ than in areas of normal and metaplastic epithelia ($P < 0.001$).

The cases were divided into six groups according to the percentage of p53-positive nuclei in the whole epithelial layer as follows: 0, negative; (+), <1% of positive nuclei; +, 1–5%; ++, 6–10%; +++, 11–40%; and ++++, >40% of positive nuclei.

### Immunohistochemistry for proliferating cell nuclear antigen (PCNA), bcl-2, bax and mcl-1

The procedure of bcl-2 immunohistochemistry using anti-human bcl-2 antibody (clone 124, Dako, Glostrup, Denmark) is described in our previous report (Törmänen et al, 1995). The immunohistochemistry for PCNA, bax and mcl-1 was performed similarly using the following antibodies: monoclonal anti-PCNA 19A2 (BioGenex, San Ramon, CA, USA) at a dilution of 1:20, incubated 30 min at room temperature; polyclonal anti-human bax (Pharmingen, San Diego, CA, USA) at a dilution of 1:1000, incubating overnight at room temperature; polyclonal anti-human mcl-1 (Pharmingen) at a dilution of 1:1000, incubating overnight at room temperature.

The positivity of bcl-2 was evaluated separately for the basal epithelial layer and the other layers, and three groups were formed based on the following classification: 0, negative; 1, positive only in basal cell layer; and 2, positive in suprabasal epithelium. The proliferative activity is defined as the percentage of PCNA-positive nuclei in the whole epithelial cell population. To describe the expression of bax and mcl-1 in the bronchial epithelium, the intensity of the immunostaining was evaluated as follows: 1, weak cytoplasmic staining; 2, moderate cytoplasmic staining; 3, strong cytoplasmic staining. Based on the quantity of the staining, the specimens were divided into three groups: 1, 1–25% of positive cells; 2, 26–50% of positive cells; 3, >50% of positive cells, for bax or mcl-1. A combined index based on both the intensity and the quantity of the immunostaining was determined by adding the qualitative and the quantitative scores and, based on this, two groups were formed: weak immunoreactivity (scores 0–4) and strong immunoreactivity (scores 4–6).

### Control stainings

A SQCLC previously shown to be p53 positive (Soini et al, 1992) was used as a positive control for p53 immunostaining. A hyperplastic lymph node was used as a control for the labelling of apoptotic DNA fragments and for bcl-2, PCNA, bax and mcl-1 immunohistochemistry. Negative controls for all immunostainings were obtained by substituting the primary antibody with PBS.

### Statistical analysis

The statistical analysis was performed with the SPSS for Windows program package (Chicago, IL, USA). The values of the apoptotic indices are reported as means with ranges. The significance of the associations was determined by using the $\chi^2$ test, Fisher's exact probability test, or Student's two-tailed *t*-test. Probability values of less than 0.05 were considered to be significant.

*© Cancer Research Campaign 1999*

**Table 3** PCNA immunoreactivity

| | Percentage of PCNA-positive nuclei | | |
|---|---|---|---|
| **Type of epithelium** | **Mean** | **Range** | **Total number of sections** |
| Normal | 1 | 0–5 | 14 |
| Squamous cell metaplasia | 6 | 1–15 | 8 |
| Mild dysplasia | 24 | 8–40 | 3 |
| Moderate dysplasia | 10 | 5–20 | 8 |
| Severe dysplasia | 23 | 16–28 | 6 |
| Carcinoma in situ | 28 | 8–40 | 5 |

**Table 4** p53 and PCNA immunoreactivity

| | PCNA immunoreactivity, number of cases | |
|---|---|---|
| **p53 status** | **≤5% Positive nuclei** | **>5% Positive nuclei** |
| Negative | 17 | 9 |
| Positive | 0 | 6 |

p53-positive nuclei were more frequently detected in areas in which over 5% of the nuclei were positive for PCNA than in areas of lower proliferation rate ($P$ = 0.006).

### RESULTS

### Apoptosis in normal bronchial epithelium and premalignant lesions

Bronchial epithelial cells in different stages of apoptosis could be easily detected after 3′ end labelling. Apoptotic cells, as well as apoptotic bodies comprising small membrane-bound fragments representing the last phase of apoptosis, were distributed throughout the whole epithelial layer in all types of epithelium studied (Figure 1A–D). The extent of apoptosis is presented in Table 1. Interestingly, the mean apoptotic indices of the metaplastic epithelium were about threefold higher than those of the normal bronchial epithelium. In mild and moderate dysplasias, the apoptotic indices were over four times higher than normal epithelium. When the samples were divided into two groups, the apoptotic indices were significantly higher in premalignant lesions (i.e. mild, moderate and severe dysplasias and carcinoma in situ) than in normal and metaplastic bronchial epithelia ($P$ = 0.001; Table 1).

### Immunoreactivity of p53 and PCNA in normal bronchial epithelium and preinvasive lesions

The distribution of p53 positivity is presented in Table 2. No positivity could be seen in normal epithelium. In dysplasias, p53-positive nuclei were mainly seen as groups throughout the epithelial thickness. Abnormal accumulation of p53 protein (>1% of p53-positive nuclei) was more often observed in mild, moderate and severe dysplasias and in carcinoma in situ than in areas of normal and metaplastic epithelia ($P$ = 0.009; Table 2).

The extent of cell proliferation, as measured by PCNA immunohistochemistry, is shown in Table 3. PCNA-positive nuclei were significantly more abundant in premalignant lesions than in normal and metaplastic bronchial epithelia ($P$ < 0.0001). p53-positive nuclei were more frequently detected in areas in which over 5% of the nuclei were positive for PCNA than in areas presenting lower proliferation rates ($P$ = 0.006; Table 4).

### Expression of bcl-2, bax and mcl-1

A uniform cytoplasmic staining pattern was seen in bcl-2-positive cells. In 13 samples of normal epithelium, bcl-2 positivity was detected in the basal cell layer. In two cases of squamous cell metaplasia, a positive reaction could be observed either in the basal cell layer or in the suprabasal layers of the epithelium. bcl-2 positivity was seen throughout the whole epithelial thickness in 7 out of 21 cases of premalignant epithelial lesions, and only in the basal cell layer in seven cases. Fourteen cases were negative for bcl-2. Data could not be obtained in one case because of depletion of tissue from the paraffin block.

Of the 18 cases showing strong immunoreactivity for bax (with a combined score, based on both the intensity and the quantity of the staining, higher than 4), ten were samples of normal or metaplastic bronchial epithelia, five were low-grade dysplasias (i.e. mild or moderate) and three were high-grade dysplasias (i.e. severe dysplasia or carcinoma in situ). Seventeen cases (eight normal or metaplastic, four low-grade and five high-grade dysplasias) showed strong immunoreactivity for mcl-1. No differences were found between the normal–metaplastic group and the premalignant group in either bax or mcl-1 immunoreactivities. Similarly, no correlation was found between the expression of bcl-2 and bax when all the samples were evaluated as a single group or in smaller groups.

### Apoptosis in relation to p53, PCNA, bcl-2, bax and mcl-1

The mean apoptotic indices of all the bronchial epithelial lesions studied were higher in p53-positive than in p53-negative lesions ($P$ = 0.009; Table 5). However, no correlation between the extent of apoptosis and positive p53 immunostaining was found when the premalignant groups were evaluated separately (Table 5). A high level of proliferative activity, as measured by PCNA immunohistochemistry, was associated with a higher apoptotic index ($P$ = 0.012). No correlation was found between apoptotic activity and the expression of bcl-2, bax and mcl-1 (Table 6).

© Cancer Research Campaign 1999 

**Table 5** Apoptotic indices and p53 expression

| Type of epithelium | p53 status | Apoptotic index (%) Mean | Range | Number of sections |
|---|---|---|---|---|
| Normal | p53 – | 0.34 | 0.00–0.69 | 14 |
| | p53 + | | | 0 |
| Squamous cell metaplasia | p53 – | 0.91 | 0.40–1.57 | 7 |
| | p53 + | 1.00 | | 1 |
| Mild dysplasia | p53 – | 1.45 | 0.68–1.94 | 3 |
| | p53 + | | | 0 |
| Moderate dysplasia | p53 – | 1.45 | 0.18–4.40 | 6 |
| | p53 + | 2.30 | 2.00–2.59 | 2 |
| Severe dysplasia | p53 – | 1.13 | 0.52–1.62 | 4 |
| | p53 + | 1.84 | 1.04–2.63 | 2 |
| Carcinoma in situ | p53 – | | | 0 |
| | p53 + | 1.49 | 0.63–3.26 | 5 |

**Table 6** Apoptotic activity and the expression of bcl-2, mcl-1 and bax. Figures represent number of cases

| | Apoptotic activity | | | | Total number of cases |
|---|---|---|---|---|---|
| | Normal and metaplastic tissue | | Premalignant tissue | | |
| | ≤0.74% | >0.74% | ≤0.74% | >0.74% | |
| bcl-2 | | | | | |
| Negative | 4 | 3 | 0 | 7 | 14 |
| Positive | 13 | 2 | 4 | 10 | 29 |
| mcl-l | | | | | |
| Index ≤4 | 2 | 1 | 0 | 5 | 8 |
| Index >4 | 8 | 0 | 3 | 6 | 17 |
| bax | | | | | |
| Index ≤4 | 3 | 0 | 1 | 6 | 10 |
| Index>4 | 9 | 1 | 1 | 7 | 18 |

### DISCUSSION

Apoptosis is one of the most actively studied phenomena in the field of cell biology at the moment. There are several reports describing its extent in neoplasias of different tissues, and most indicate that apoptosis is more frequent in malignant than in normal tissue (Lipponen and Aaltomaa, 1994; Aihara et al, 1995; Arai and Kino, 1995; Bardeesy et al, 1995; Staunton and Gaffney, 1995; Törmänen et al, 1995; Soini et al, 1996). However, studies of preneoplastic lesions are rare and, to our knowledge, none have been published on bronchial dysplasias. The main purpose of the present work was to study the extent of apoptosis in normal and metaplastic bronchial epithelia and in non-invasive, premalignant lesions preceding SQCLC, i.e. mild, moderate and severe dysplasias and squamous cell carcinoma in situ.

We found the relative number of apoptotic cells and bodies (apoptotic index) to increase as the normal epithelium of the bronchus gradually alters to a premalignant lesion. In normal epithelium, single cells undergoing apoptosis were seldom seen. We assume that, in the normal epithelium, the apoptotic programme functions normally, eliminating cells with a genetic defect. Hence, an increased number of apoptotic cells and bodies in metaplastic and dysplastic lesions might be the result of normal activity, rather than of an impaired expression of apoptosis-regulating genes, such as *p53*, thus reflecting a larger number of damaged cells in the lesion.

In line with some previous studies, we found aberrant p53 expression to be associated with the severity of the dysplasia (Sundaresan et al, 1992; Bennett et al, 1993; Nuorva et al, 1993; Hirano et al, 1994; Walker et al, 1994). We also found the p53 protein to accumulate in lesions preceding SQCLC, starting with mild dysplasia. The apoptosis-inducing function of *p53* has been thought to vanish whenever mutations occur (Lowe et al, 1994; Bardeesy et al, 1995). This hypothesis is supported by the results from Wilms' tumours (Bardeesy et al, 1995) and small-cell lung carcinomas (Eerola et al, 1997), in which there was an inverse correlation between the extent of apoptosis and *p53* mutations or p53 immunoreactivity. In contrast, Lipponen and Aaltomaa (1994) demonstrated that p53-positive bladder tumours show a significantly higher number of apoptotic cells and bodies than p53-negative tumours. In non-small-cell lung carcinomas, we found no

 *© Cancer Research Campaign 1999*

correlation between these two parameters (Törmänen et al, 1995). In this study, both the apoptotic index and the degree of p53 protein accumulation increased with the severity of the dysplasia. These strikingly different results on the relationship between p53 expression and apoptosis in different types of carcinoma emphasize the need to study the effects of different mutations on the apoptosis-regulating characteristics of the *p53* gene. The study of the expression of other apoptosis-regulating genes in different tissues would also be of value because the lost ability of *p53* to induce apoptosis might be compensated by other, more powerful regulators. One might also hypothesize that the elevation of the apoptotic indices in bronchial dysplasias is due to a normal, apoptosis-promoting function of *p53*. In our opinion, this theory is contradicted by the observation that the accumulation of immunohistochemically detectable p53 protein increased in parallel with the severity of the dysplasia. The p53 protein in the lesions would thus be mutated, or otherwise inactivated, and unable to induce apoptosis.

The results of this study indicate that both the rate of cell proliferation and the quantity of cell death are altered in parallel with the morphological changes. We found a direct correlation between the extent of apoptosis and the rate of cell proliferation, as evaluated by PCNA immunohistochemistry. A similar association has been previously shown in colorectal tubular and villous adenomas (Arai and Kino, 1995) and carcinomas (Baretton et al, 1996), as well as in gastric (Koshida et al, 1997), endometrial (Saegusa et al, 1996), breast (Lipponen et al, 1994) and bladder (King et al, 1996) carcinomas.

The expression of bcl-2 in this selection of normal bronchial epithelium and dysplasias of different severity was quite similar to that reported by Walker et al (1995). In our work, however, no increase of bcl-2 expression could be seen as the severity of dysplasia increased. Previously, we found no correlation between the extent of apoptosis and the expression of bcl-2 in non-small-cell lung carcinomas (Törmänen et al, 1995). Similarly, no association between these two factors could be found in bronchial dysplasias. Furthermore, no correlation was found between apoptosis and the expression of bax and mcl-1.

The independence of apoptosis from the expression of p53 and the bcl-2 family proteins suggests that the apoptotic signalling pathway in bronchial dysplasias functions independently of these factors. As it has been shown that the *Fas* receptor is expressed in lung tumours (Hellquist et al, 1997), the main pathway leading to apoptosis in bronchial dysplasias preceding SQCLC could also include the activation of the *Fas* receptor. In this system, the *Fas* ligand, bound for example to the cytotoxic lymphocytes, binds to the oligomerized *Fas* receptor which then, via FADD-MORT, activates FLICE (i.e. caspase 8) and, thus, the caspase cascade, leading to apoptosis without the involvement of *p53* and the bcl-2-related proteins (Muzio et al, 1996; Nagata, 1997). This could explain the independence of apoptosis from both the expression of p53 and of the gene products of the *bcl-2* family in bronchial dysplasias and NSCLC.

Based on these results, it seems that the elevation of the apoptotic index is an early event in the process in which the normal bronchial epithelium changes to squamous cell carcinoma in situ, and that the increase in the apoptotic activity is associated with the severity of the bronchial premalignant lesion, i.e. dysplasia. The highest apoptotic indices were found in severe dysplasias and carcinoma in situ, exceeding even those reported for SQCLC in our previous study (Törmänen et al, 1995). It is possible that the high apoptotic activity in a premalignant lesion reflects an attempt to eliminate genetically damaged cells and that, in the following invasive carcinoma, the aggregated mutations somehow interfere with the apoptosis-regulating mechanisms. In line with our observations, Birchall et al (1995) and Ishida et al (1996) have demonstrated that the apoptotic activity is higher in premalignant gastric lesions and in dysplasias of the oral cavity than in the corresponding invasive carcinomas.

### ACKNOWLEDGEMENTS

This study was supported by the Finnish Cancer Societies and the Finnish Anti-Tuberculosis Association Foundation.

### REFERENCES

Aihara M, Scardino PT, Truong LD, Wheeler TM, Goad JR, Yang G and Thompson TC (1995) The frequency of apoptosis correlates with the prognosis of Gleason grade 3 adenocarcinoma of the prostate. *Cancer* **75**: 522–529

Arai T and Kino I (1995) Role of apoptosis in modulation of the growth of human colorectal tubular and villous adenomas. *J Pathol* **176**: 37–44

Auerbach O, Gere B, Forman J, Petrick T, Smolin H, Muehsam G, Kassouny D and Stout A (1957) Changes in the bronchial epithelium in relation to smoking and cancer of the lung. *N Engl J Med* **256**: 97–104

Auerbach O, Stout A, Hammond E and Garfinkel L (1962) Changes in bronchial epithelium in relation to sex, age, residence, smoking and pneumonia. *N Engl J Med* **267**: 111–125

Auerbach O, Hammond E and Garfinkel L (1979) Changes in bronchial epithelium in relation to cigarette smoking, 1955–1960 vs. 1970–1977. *N Engl J Med* **300**: 381–386

Bakhshi A, Jensen JP, Goldman P, Wright JJ, Wesley McBride O, Epstein, AL and Korsmeyer SJ (1985) Cloning of the chromosomal breakpoint of t(14:18) human lymphomas: clustering around $J_H$ on chromosome 14 and near a transcriptional unit on chromosome 18. *Cell* **41**: 899–906

Bardeesy N, Beckwith JB and Pelletier J (1995) Clonal expansion and attenuated apoptosis in Wilms' tumors are associated with p53 gene mutations. *Cancer Res* **55**: 215–219

Baretton GB, Diebold J, Christoforis G, Vogt M, Müller C, Dopfer K, Schneiderbanger K, Schmidt M and Löhrs U (1996) Apoptosis and immunohistochemical bcl-2 expression in colorectal adenomas and carcinomas. Aspects of carcinogenesis and prognostic significance. *Cancer* **77**: 255–264

Bennett WP, Colby TV, Travis WD, Borkowski A, Jones RT, Lane DP, Metcalf RA, Samet JM, Takeshima Y, Gu JR, Vähäkangas KV, Soini Y, Pääkkö P, Welsh JA, Trump BF and Harris CC (1993) p53 protein accumulates frequently in early bronchial neoplasia. *Cancer Res* **53**: 4817–4822

Birchall MA, Winterford CM, Allan DJ and Harmon BV (1995) Apoptosis in normal epithelium, premalignant and malignant lesions of the oropharynx and oral cavity: a preliminary study. *Eur J Cancer Oral Oncol* **31B**: 380–383

Bodrug SE, Aimé-Sempé C, Sato T, Krajewski S, Hanada M and Reed JC (1995) Biochemical and functional comparisons of Mcl-1 and Bcl-2 proteins: evidence for a novel mechanism of regulating Bcl-2 family protein function. *Cell Death Differ* **2**: 173–182

Eerola A-K, Törmänen U, Rainio P, Vähäkangas K, Sormunen R, Bloigu R, Lehto V-P and Pääkkö P (1997) Apoptosis in operated small cell lung carcinoma is inversely related to tumour necrosis and p53 immunoreactivity. *J Pathol* **181**: 172–177

Greenblatt MS, Bennett WP, Hollstein M and Harris CC (1994) Mutations in the p53 tumor suppressor gene: clues to cancer etiology and molecular pathogenesis. *Cancer Res* **54**: 4855–4878

Hellquist HB, Olejnicka B, Jadner M, Andersson T and Sederholm C (1997) Fas receptor is expressed in human lung squamous cell carcinomas, whereas bcl-2 and apoptosis are not pronounced: a preliminary report. *Br J Cancer* **76**: 175–179

Hirano T, Franzén B, Kato H, Ebihara Y and Auer G (1994) Genesis of squamous cell lung carcinoma. Sequential changes of proliferation, DNA ploidy and p53 expression. *Am J Pathol* **144**: 296–302

Hockenbery DM, Zutter M, Hickey W, Nahm M and Korsmeyer SJ (1991) Bcl-2 protein is topographically restricted in tissues characterized by apoptotic cell death. *Proc Natl Acad Sci USA* **88**: 6961–6965

Ishida M, Gomyo Y, Tatebe S, Ohfuji S and Ito H (1996) Apoptosis in human gastric mucosa, chronic gastritis, dysplasia and carcinoma: analysis by terminal deoxynucleotidyl transferase-mediated dUTP-biotin nick end labelling. *Virchows Arch* **428**: 229–235

Kerr JFR, Wyllie AH and Currie AR (1972) Apoptosis: a basic biological phenomenon with wide-ranging implications in tissue kinetics. *Br J Cancer* **26**: 239–257

King ED, Matteson J, Jacobs SC and Kyprianou N (1996) Incidence of apoptosis, cell proliferation and bcl-2 expression in transitional cell carcinoma of the bladder: association with tumor progression. *J Urol* **155**: 316–320

Lipponen PK and Aaltomaa S (1994) Apoptosis in bladder cancer as related to standard prognostic factors and prognosis. *J Pathol* **173**: 333–339

Lipponen P, Aaltomaa S, Kosma V-M and Syrjänen K (1994) Apoptosis in breast cancer as related to histopathological characteristics and prognosis. *Eur J Cancer* **14**: 2068–2073

Lowe SW, Bodis S, McClatchey A, Remington L, Ruley HE, Fisher DE, Housman DE and Jacks T (1994) p53 status and the efficacy of cancer therapy in vivo. *Science* **266**: 807–810

Koshida Y, Saegusa M and Okayasu I (1997) Apoptosis, cell proliferation and expression of bcl-2 and bax in gastric carcinomas: immunohistochemical and clinicopathological study. *Br J Cancer* **75**: 367–373

Kroemer G (1997) The proto-oncogene Bcl-2 and its role in regulating apoptosis. *Nature Med* **6**: 614–620

Lu, Q-L, Poulsom R, Wong L and Hanby AM (1993) bcl-2 expression in adult and embryonic non-haematopoietic tissues. *J Pathol* **169**: 431–437

Minn AJ, Vélez P, Schendel SL, Liang H, Muchmore SW, Fesik SW, Fill M and Thompson GB (1997) Bcl-$x_L$ forms an ion channel in synthetic lipid membranes. *Nature* **385**: 353–357

Muzio M, Chinnaiyan AM, Kischkel FC, O'Rourke K, Schevchenko A, Ni J, Scaffidi C, Bretz JD, Zhang M, Gentz R, Mann M, Krammer PH, Peter ME and Dixit VM (1996) FLICE, a novel FADD-homologous ICE/CED-3-like protease, is required to the CD95 (Fas/APO-1) death-inducing signaling complex. *Cell* **85**: 817–827

Nagata S (1997) Apoptosis by death factor. *Cell* **88**: 355–365

Nuorva K, Soini Y, Kamel D, Autio-Harmainen H, Risteli L, Risteli J, Vähäkangas K and Pääkkö P (1993) Concurrent p53 expression in bronchial dysplasias and squamous cell lung carcinomas. *Am J Pathol* **142**: 725–732

Oltvai Z, Milliman C and Korsmeyer SJ (1993) Bcl-2 heterodimerizes in vivo with a conserved homolog, Bax, that accelerates programmed cell death. *Cell* **74**: 609–619

Reed JC (1994) Bcl-2 and the regulation of programmed cell death. *J Cell Biol* **124**: 1–6

Saegusa M, Kamata Y, Isono M and Okayasu I (1996) bcl-2 expression is correlated with a low apoptotic index and is associated with progesterone receptor immunoreactivity in endometrial carcinomas. *J Pathol* **180**: 275–282

Soini Y, Kamel D, Nuorva K, Lane DP, Vähäkangas K and Pääkkö P (1992) Low p53 protein expression in salivary gland tumours compared with lung carcinomas. *Virchows Arch (Pathol Anat)* **421**: 415–420

Soini Y, Virkajärvi N, Lehto V-P and Pääkkö P (1996) Hepatocellular carcinomas with a high proliferation index and a low degree of apoptosis and necrosis are associated with a shortened survival. *Br J Cancer* **73**: 1025–1030

Sozzi G, Miozzo M, Donghi R, Pilotti S, Cariani CT, Pastorino U, Della Porta G and Pierotti MA (1992) Deletions of 17p and p53 mutations in preneoplastic lesions of the lung. *Cancer Res* **52**: 6079–6082

Staunton MJ and Gaffney EF (1995) Tumor type is a determinant of susceptibility to apoptosis. *Am J Clin Pathol* **103**: 300–307

Sundaresan V, Ganly P, Hasleton P, Rudd R, Sinha G, Bleehen NM and Rabbits P (1992) p53 and chromosome 3 abnormalities, characteristic of malignant lung tumours, are detectable in preinvasive lesions of the bronchus. *Oncogene* **7**: 1989–1997

Törmänen U, Eerola A-K, Rainio P, Vähäkangas K, Soini Y, Sormunen R, Bloigu R, Lehto V-P and Pääkkö P (1995) Enhanced apoptosis predicts shortened survival in non-small cell lung carcinoma. *Cancer Res* **55**: 5595–5602

Tsujimoto Y, Finger LR, Yunis J, Nowell PC and Croce CM (1984) Cloning of the chromosome breakpoint of neoplastic B cells with the t(14:18) chromosome translocation. *Science* **226**: 1097–1099

Vähäkangas KH, Samet JM, Metcalf RA, Welsh JA, Bennett WP, Lane DP and Harris CC (1992) Mutations of p53 and ras genes in radon-associated lung cancer from uranium miners. *Lancet* **339**: 576–580

Walker C, Robertson LJ, Myskow MW, Pendleton N and Dixon GR (1994) p53 expression in normal and dysplastic bronchial epithelium and in lung carcinomas. *Br J Cancer* **70**: 297–303

Walker C, Robertson L, Myskow M and Dixon G (1995) Expression of the bcl-2 protein in normal and dysplastic bronchial epithelium and in lung carcinomas. *Br J Cancer* **72**: 164–169

World Health Organization (1981) *Histological Typing of Lung Tumours*. International Classification of Tumours. No 1. World Health Organization: Geneva

Yang J, Xuesong L, Bhalla K, Caryn Naekyung K, Ibrado AM, Cai J, Tsung-IP, Jones DP and Wang X (1997) Prevention of apoptosis by bcl-2: release of cytochrome c from mitochondria blocked. *Science* **275**: 1129–1132

*© Cancer Research Campaign 1999*

## References

[PDF_00463] AUERBACH O., FORMAN J. B., GERE J. B., KASSOUNY D. Y., MUEHSAM G. E., PETRICK T. G., SMOLIN H. J., STOUT A. P. (1957). Changes in the bronchial epithelium in relation to smoking and cancer of the lung; a report of progress.. N Engl J Med.

[PDF_00466] AUERBACH O., STOUT A. P., HAMMOND E. C., GARFINKEL L. (1962). Changes in bronchial epithelium in relation to sex, age, residence, smoking and pneumonia.. N Engl J Med.

[PDF_00458] Aihara M., Scardino P. T., Truong L. D., Wheeler T. M., Goad J. R., Yang G., Thompson T. C. (1995). The frequency of apoptosis correlates with the prognosis of Gleason Grade 3 adenocarcinoma of the prostate.. Cancer.

[PDF_00461] Arai T., Kino I. (1995). Role of apoptosis in modulation of the growth of human colorectal tubular and villous adenomas.. J Pathol.

[PDF_00469] Auerbach O., Hammond E. C., Garfinkel L. (1979). Changes in bronchial epithelium in relation to cigarette smoking, 1955-1960 vs. 1970-1977.. N Engl J Med.

[PDF_00472] Bakhshi A., Jensen J. P., Goldman P., Wright J. J., McBride O. W., Epstein A. L., Korsmeyer S. J. (1985). Cloning the chromosomal breakpoint of t(14;18) human lymphomas: clustering around JH on chromosome 14 and near a transcriptional unit on 18.. Cell.

[PDF_00477] Bardeesy N., Beckwith J. B., Pelletier J. (1995). Clonal expansion and attenuated apoptosis in Wilms' tumors are associated with p53 gene mutations.. Cancer Res.

[PDF_00480] Baretton G. B., Diebold J., Christoforis G., Vogt M., Müller C., Dopfer K., Schneiderbanger K., Schmidt M., Löhrs U. (1996). Apoptosis and immunohistochemical bcl-2 expression in colorectal adenomas and carcinomas. Aspects of carcinogenesis and prognostic significance.. Cancer.

[PDF_00485] Bennett W. P., Colby T. V., Travis W. D., Borkowski A., Jones R. T., Lane D. P., Metcalf R. A., Samet J. M., Takeshima Y., Gu J. R. (1993). p53 protein accumulates frequently in early bronchial neoplasia.. Cancer Res.

[PDF_00489] Birchall M. A., Winterford C. M., Allan D. J., Harmon B. V. (1995). Apoptosis in normal epithelium, premalignant and malignant lesions of the oropharynx and oral cavity: a preliminary study.. Eur J Cancer B Oral Oncol.

[PDF_00492] Bodrug S. E., Aimé-Sempé C., Sato T., Krajewski S., Hanada M., Reed J. C. (1995). Biochemical and functional comparisons of Mcl-1 and Bcl-2 proteins: evidence for a novel mechanism of regulating Bcl-2 family protein function.. Cell Death Differ.

[PDF_00496] Eerola A. K., Törmänen U., Rainio P., Sormunen R., Bloigu R., Vähäkangas K., Lehto V. P., Soini Y., Päkkö P. (1997). Apoptosis in operated small cell lung carcinoma is inversely related to tumour necrosis and p53 immunoreactivity.. J Pathol.

[PDF_00500] Greenblatt M. S., Bennett W. P., Hollstein M., Harris C. C. (1994). Mutations in the p53 tumor suppressor gene: clues to cancer etiology and molecular pathogenesis.. Cancer Res.

[PDF_00503] Hellquist H. B., Olejnicka B., Jadner M., Andersson T., Sederholm C. (1997). Fas receptor is expressed in human lung squamous cell carcinomas, whereas bcl-2 and apoptosis are not pronounced: a preliminary report.. Br J Cancer.

[PDF_00507] Hirano T., Franzén B., Kato H., Ebihara Y., Auer G. (1994). Genesis of squamous cell lung carcinoma. Sequential changes of proliferation, DNA ploidy, and p53 expression.. Am J Pathol.

[PDF_00510] Hockenbery D. M., Zutter M., Hickey W., Nahm M., Korsmeyer S. J. (1991). BCL2 protein is topographically restricted in tissues characterized by apoptotic cell death.. Proc Natl Acad Sci U S A.

[PDF_00515] Ishida M., Gomyo Y., Tatebe S., Ohfuji S., Ito H. (1996). Apoptosis in human gastric mucosa, chronic gastritis, dysplasia and carcinoma: analysis by terminal deoxynucleotidyl transferase-mediated dUTP-biotin nick end labelling.. Virchows Arch.

[PDF_00519] Kerr J. F., Wyllie A. H., Currie A. R. (1972). Apoptosis: a basic biological phenomenon with wide-ranging implications in tissue kinetics.. Br J Cancer.

[PDF_00522] King E. D., Matteson J., Jacobs S. C., Kyprianou N. (1996). Incidence of apoptosis, cell proliferation and bcl-2 expression in transitional cell carcinoma of the bladder: association with tumor progression.. J Urol.

[PDF_00533] Koshida Y., Saegusa M., Okayasu I. (1997). Apoptosis, cell proliferation and expression of Bcl-2 and Bax in gastric carcinomas: immunohistochemical and clinicopathological study.. Br J Cancer.

[PDF_00536] Kroemer G. (1997). The proto-oncogene Bcl-2 and its role in regulating apoptosis.. Nat Med.

[PDF_00525] Lipponen P. K., Aaltomaa S. (1994). Apoptosis in bladder cancer as related to standard prognostic factors and prognosis.. J Pathol.

[PDF_00527] Lipponen P., Aaltomaa S., Kosma V. M., Syrjänen K. (1994). Apoptosis in breast cancer as related to histopathological characteristics and prognosis.. Eur J Cancer.

[PDF_00530] Lowe S. W., Bodis S., McClatchey A., Remington L., Ruley H. E., Fisher D. E., Housman D. E., Jacks T. (1994). p53 status and the efficacy of cancer therapy in vivo.. Science.

[PDF_00538] Lu Q. L., Poulsom R., Wong L., Hanby A. M. (1993). Bcl-2 expression in adult and embryonic non-haematopoietic tissues.. J Pathol.

[PDF_00540] Minn A. J., Vélez P., Schendel S. L., Liang H., Muchmore S. W., Fesik S. W., Fill M., Thompson C. B. (1997). Bcl-x(L) forms an ion channel in synthetic lipid membranes.. Nature.

[PDF_00544] Muzio M., Chinnaiyan A. M., Kischkel F. C., O'Rourke K., Shevchenko A., Ni J., Scaffidi C., Bretz J. D., Zhang M., Gentz R. (1996). FLICE, a novel FADD-homologous ICE/CED-3-like protease, is recruited to the CD95 (Fas/APO-1) death--inducing signaling complex.. Cell.

[PDF_00549] Nagata S. (1997). Apoptosis by death factor.. Cell.

[PDF_00550] Nuorva K., Soini Y., Kamel D., Autio-Harmainen H., Risteli L., Risteli J., Vähäkangas K., Päkkö P. (1993). Concurrent p53 expression in bronchial dysplasias and squamous cell lung carcinomas.. Am J Pathol.

[PDF_00553] Oltvai Z. N., Milliman C. L., Korsmeyer S. J. (1993). Bcl-2 heterodimerizes in vivo with a conserved homolog, Bax, that accelerates programmed cell death.. Cell.

[PDF_00556] Reed J. C. (1994). Bcl-2 and the regulation of programmed cell death.. J Cell Biol.

[PDF_00558] Saegusa M., Kamata Y., Isono M., Okayasu I. (1996). Bcl-2 expression is correlated with a low apoptotic index and associated with progesterone receptor immunoreactivity in endometrial carcinomas.. J Pathol.

[PDF_00562] Soini Y., Kamel D., Nuorva K., Lane D. P., Vähäkangas K., Päkkö P. (1992). Low p53 protein expression in salivary gland tumours compared with lung carcinomas.. Virchows Arch A Pathol Anat Histopathol.

[PDF_00565] Soini Y., Virkajärvi N., Lehto V. P., Päkkö P. (1996). Hepatocellular carcinomas with a high proliferation index and a low degree of apoptosis and necrosis are associated with a shortened survival.. Br J Cancer.

[PDF_00568] Sozzi G., Miozzo M., Donghi R., Pilotti S., Cariani C. T., Pastorino U., Della Porta G., Pierotti M. A. (1992). Deletions of 17p and p53 mutations in preneoplastic lesions of the lung.. Cancer Res.

[PDF_00571] Staunton M. J., Gaffney E. F. (1995). Tumor type is a determinant of susceptibility to apoptosis.. Am J Clin Pathol.

[PDF_00573] Sundaresan V., Ganly P., Hasleton P., Rudd R., Sinha G., Bleehen N. M., Rabbitts P. (1992). p53 and chromosome 3 abnormalities, characteristic of malignant lung tumours, are detectable in preinvasive lesions of the bronchus.. Oncogene.

[PDF_00581] Tsujimoto Y., Finger L. R., Yunis J., Nowell P. C., Croce C. M. (1984). Cloning of the chromosome breakpoint of neoplastic B cells with the t(14;18) chromosome translocation.. Science.

[PDF_00577] Törmänen U., Eerola A. K., Rainio P., Vähäkangas K., Soini Y., Sormunen R., Bloigu R., Lehto V. P., Päkkö P. (1995). Enhanced apoptosis predicts shortened survival in non-small cell lung carcinoma.. Cancer Res.

[PDF_00584] Vähäkangas K. H., Samet J. M., Metcalf R. A., Welsh J. A., Bennett W. P., Lane D. P., Harris C. C. (1992). Mutations of p53 and ras genes in radon-associated lung cancer from uranium miners.. Lancet.

[PDF_00587] Walker C., Robertson L. J., Myskow M. W., Pendleton N., Dixon G. R. (1994). p53 expression in normal and dysplastic bronchial epithelium and in lung carcinomas.. Br J Cancer.

[PDF_00590] Walker C., Robertson L., Myskow M., Dixon G. (1995). Expression of the BCL-2 protein in normal and dysplastic bronchial epithelium and in lung carcinomas.. Br J Cancer.

[PDF_00596] Yang J., Liu X., Bhalla K., Kim C. N., Ibrado A. M., Cai J., Peng T. I., Jones D. P., Wang X. (1997). Prevention of apoptosis by Bcl-2: release of cytochrome c from mitochondria blocked.. Science.

